# Opportunities in posthemorrhagic hydrocephalus research: outcomes of the Hydrocephalus Association Posthemorrhagic Hydrocephalus Workshop

**DOI:** 10.1186/s12987-018-0096-3

**Published:** 2018-03-27

**Authors:** Jenna E. Koschnitzky, Richard F. Keep, David D. Limbrick, James P. McAllister, Jill A. Morris, Jennifer Strahle, Yun C. Yung

**Affiliations:** 1 Hydrocephalus Association, 4340 East West Highway, Bethesda, MD 20814 USA; 2 University of Michigan, 1500 East Medical Center Drive, Ann Arbor, MI 48109 USA; 3 Washington University in St. Louis School of Medicine, 660 S. Euclid Ave, St. Louis, MO 63110 USA; 4 National Institute of Neurological Disorders and Stroke, National Institutes of Health, Neuroscience Center, 6001 Executive Blvd, NSC Rm 2112, Bethesda, MD 20892 USA; 5 Sanford Burnham Prebys Medical Discovery Institute, 10901 North Torrey Pines Rd., Building 7, La Jolla, CA 92037 USA

**Keywords:** Hydrocephalus, Posthemorrhagic, Intraventricular hemorrhage, Germinal matrix hemorrhage, Blood brain barrier, Premature

## Abstract

The Hydrocephalus Association Posthemorrhagic Hydrocephalus Workshop was held on July 25 and 26, 2016 at the National Institutes of Health. The workshop brought together a diverse group of researchers including pediatric neurosurgeons, neurologists, and neuropsychologists with scientists in the fields of brain injury and development, cerebrospinal and interstitial fluid dynamics, and the blood–brain and blood–CSF barriers. The goals of the workshop were to identify areas of opportunity in posthemorrhagic hydrocephalus research and encourage scientific collaboration across a diverse set of fields. This report details the major themes discussed during the workshop and research opportunities identified for posthemorrhagic hydrocephalus. The primary areas include (1) preventing intraventricular hemorrhage, (2) stopping primary and secondary brain damage, (3) preventing hydrocephalus, (4) repairing brain damage, and (5) improving neurodevelopment outcomes in posthemorrhagic hydrocephalus.

## Background

The Hydrocephalus Association hosted a workshop on July 25 and 26, 2016 at the National Institutes of Health, Neuroscience Center, to build research capacity and identify areas of opportunity for research around posthemorrhagic hydrocephalus (PHH). The Hydrocephalus Association was founded in 1983 by parents of children with hydrocephalus and developed a research program in 2009. Since then the Association has committed over $7 million to research, making it the largest non-profit, non-governmental funder of hydrocephalus research in the United States. The mission of the Hydrocephalus Association is to promote a cure for hydrocephalus and improve the lives of those affected by the condition. The Association strives to accomplish this mission by collaborating with patients, caregivers, researchers and industry, raising awareness, and funding innovative, high impact research to prevent, treat, and ultimately cure hydrocephalus.

The 2016 Hydrocephalus Association PHH Workshop was developed through the Hydrocephalus Association PHH Initiative, a multiyear initiative focused on increasing research efforts focused on PHH. The workshop agenda was developed by the workshop planning committee which was led by Hydrocephalus Association staff and included both an investigator in the Hydrocephalus Clinical Research Network (HCRN) and a family member of a person with hydrocephalus. The workshop brought together a diverse group of researchers including pediatric neurosurgeons, neurologists, and neuropsychologists together with scientists in the fields of brain injury and development, cerebrospinal and interstitial fluid dynamics, and the blood–brain and blood–CSF barriers. Workshop attendees also included Hydrocephalus Association staff and family members of people affected by hydrocephalus.

**Fig. 1:**
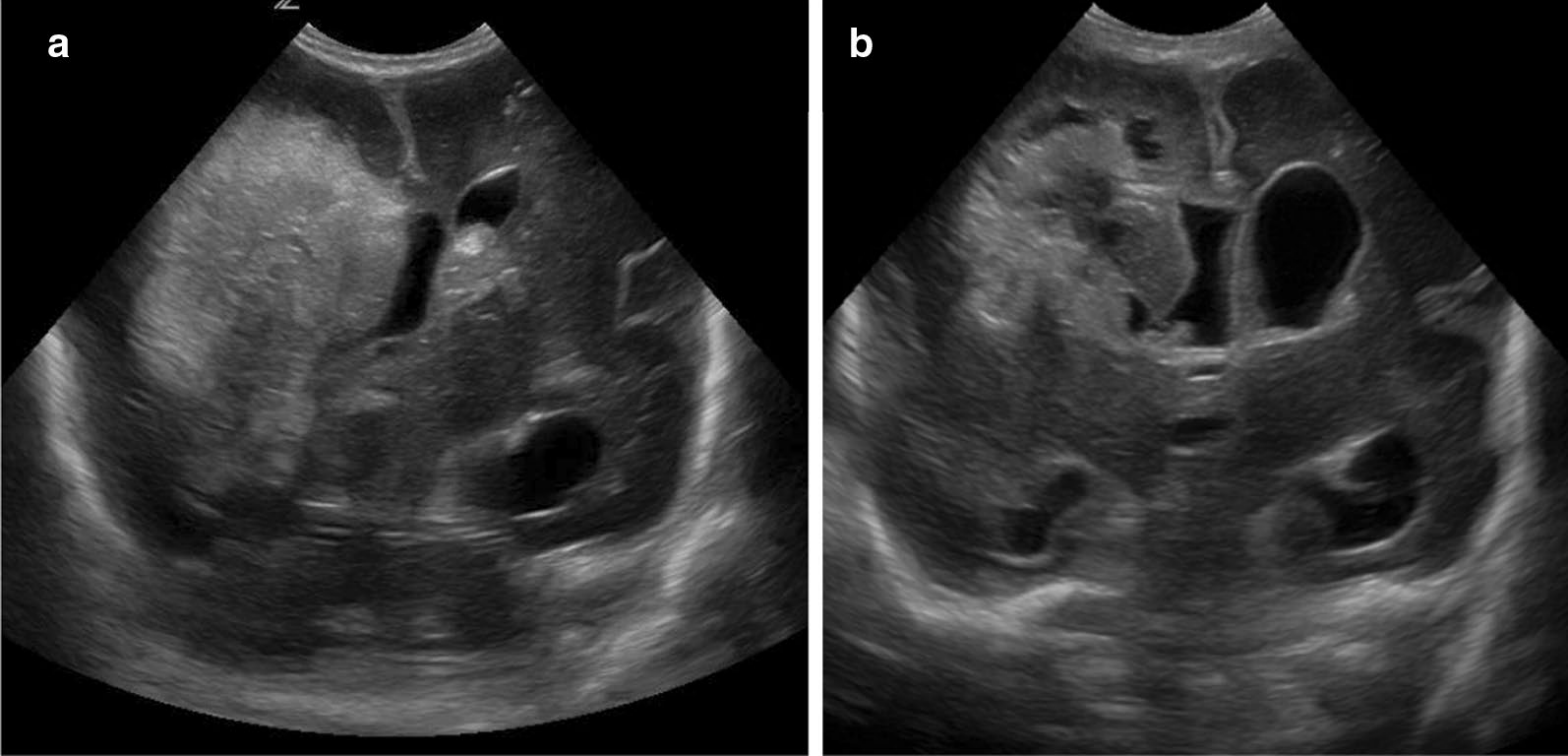
Cranial Ultrasounds of Premature Infant after IVH. **a** Cranial ultrasound performed in a 5 day old 24 week gestation male showing grade IV intraventricular hemorrhage with intraventricular extension. **b** Cranial ultrasound performed in the same infant 12 days later showing interval development of hydrocephalus

The primary focus of the workshop was on PHH of prematurity, although PHH can develop at any age, including in utero, after preterm birth, and in children and adults who have had a brain hemorrhage [1]. PHH develops after intraventricular extension of a hemorrhage [intraventricular hemorrhage, (IVH)] (Fig. 1). IVH grade (I–IV) is classified according to the extension (periventricular germinal tissue, ventricle, parenchyma) of hemorrhage and presence of ventricular dilation according to the Papile scale [2]. Both the source of the hemorrhage (e.g. periventricular vs. choroid plexus) and the degree of tissue injury related to the extension of the hemorrhage are linked to outcomes. Neonatal IVH occurs in up to 25% of preterm neonates [3–12] and results in hydrocephalus in 10% of these neonates, including 25% of neonates with grade III/IV IVH [13]. 38% of neonates with PHH require long term CSF diversion; the remaining either die or do not require a permanent shunt [13].

Advances in neonatology have improved survival rates for preterm neonates in recent decades [14]. In the early 1980 s, the rates of IVH decreased from 40–50 to 20% for premature neonates < 1500 g [15, 16]. However, for premature neonates 500–750 g, the rate of IVH remained high at 40–50% [15, 17, 18], and the trend in recent years shows a relative increase in IVH rates [13, 19, 20]. In the United States, the incidence of PHH of prematurity after IVH remained steady (8–10%) between 2000 and 2010; however, annual admissions for both IVH and PHH increased approximately 60% during the same time period [13]. The increase in IVH and PHH comes despite a recent decline in overall premature birth rate between 2007 and 2010 [13, 21].

PHH of prematurity is a common form of pediatric hydrocephalus, accounting for 20% of the shunted hydrocephalus cases in the United States [22] and is associated with significant morbidity and mortality [13]. This report details the major themes and the research opportunities discussed during the 2016 Hydrocephalus Association Posthemorrhagic Hydrocephalus Workshop. The views and recommendations in this report were developed by the listed authors and subsequently distributed to all workshop attendees for commentary. All feedback was then discussed and integrated into the report.

## PHH of prematurity

PHH of prematurity is a common and particularly insidious form of pediatric hydrocephalus [22]. During the workshop, an overview of our current knowledge about PHH risks, pathophysiology, and treatments was presented.

### Maternal and neonatal risk and protective factors

Many maternal factors can alter the risk of IVH in premature neonates. Some protective factors are related to health care including access to prenatal care, administration of antenatal corticosteroid regimens, and cesarean delivery [23]. Maternal health care disparities likely interact with other factors such as maternal race and education level which have also been associated with risk of IVH in preterm neonates. Unexpectedly, preeclampsia has been associated with a decreased risk of IVH [23–28].

Neonates born at term rarely develop IVH or PHH [29]. For premature neonates, the risk of IVH, and subsequently PHH, increases with decreased gestational age and weight [7, 13, 14]. Furthermore, the development of PHH is directly related to IVH grade, with over 75% of PHH patients having Grade III/IV IVH [13]. Male sex is also associated with an increased risk of PHH. Prenatal infection, specifically chorioamnionitis, can increase the risk of IVH and is related to gestational age [5, 23, 30–35]. Asian ethnicity seems to be protective [36].

### Physiological risk factors

Premature neonates are especially vulnerable to IVH within the subependymal germinal matrix, a highly vascularized region consisting of neural and glial precursor cells [19, 30, 37, 38]. During early development, the germinal matrix undergoes rapid cellular proliferation and angiogenesis, but the thickness of the germinal matrix peaks and subsequently declines after 24 gestational weeks and is nearly absent by 36–37 gestational weeks [3, 19, 39–41]. This corresponds to the time-dependent risk of developing IVH and subsequent PHH. At developmental time points when premature birth is survivable, the germinal matrix is located along the walls of the lateral ventricles, primarily within the ganglionic eminence and over the developing caudate nucleus.

In preterm neonates, the germinal matrix is susceptible to hemorrhaging due to the immaturity of the vasculature, [3, 15, 19, 39, 42, 43] and sudden fluctuations in cerebral blood flow due to respiratory and hemodynamic instability [37, 38, 44]. The combination of immature vasculature and rapid changes in cerebral blood flow may lead to rupture of the germinal matrix vasculature and IVH [3, 19, 30].

### Pathophysiology of PHH

Hydrocephalus represents an alteration in fluid balance within the brain and cranial cavity. It is traditionally held that under normal conditions cerebrospinal fluid (CSF) is primarily secreted into the cerebral ventricles by the choroid plexus (site of the blood–CSF barrier) and moves via bulk flow through the ventricular system and subarachnoid space before being absorbed at the arachnoid villi/granulations although it should be noted that these develop at about week 35 and 39, respectively [45]. In addition, some fluid secretion into brain parenchyma occurs across the cerebral microvasculature (site of the blood–brain barrier) and through Virchow-Robins spaces (also recently termed the “glymphatic” system) at the surface of the cerebral cortex in animal models [46–48]. Parenchymal interstitial fluid also enters the cerebral ventricles across the ependymal lining [49]. CSF can also be absorbed via cranial nerve lymphatics [50–52] and may pass retrogradely into cerebral microvessels [53–57]. An increase in CSF production or reduction in CSF absorption may result in ventriculomegaly if the system cannot compensate for the changes.

Secondary injury mechanisms occur following ventriculomegaly (reviewed in [58–60]). A detailed discussion of these secondary mechanisms is beyond the scope of this manuscript, but they include cerebral (mostly periventricular) edema, demyelination, axonal degeneration and impaired axoplasmic transport in periventricular white matter, cerebral hypoxia and ischemia, reduced metabolite levels, altered blood–brain and blood–CSF barriers, loss of cilia and junctional complexes on ependymal cells, ependymal denudation, changes in aquaporin channels, and altered neurodevelopmental trophic factors. These cytopathologies are found in most forms of congenital and acquired hydrocephalus and most likely overlap with the pathogenetic mechanisms involved in PHH.

As detailed above, the pathophysiology of hydrocephalus is multifactorial in nature, and the precise mechanisms underlying PHH of prematurity remain unclear [3]. PHH is traditionally thought to occur due to impediments in the CSF flow or absorption pathways. Acute, obstructive hydrocephalus can occur soon after IVH and may be due to blood physically blocking the most-narrow portions of the CSF spaces, i.e. the cerebral aqueduct, the foramina of Monro, the outlets of the fourth ventricle, or the subarachnoid spaces.

Multiple theories have been proposed to explain the development of “communicating” hydrocephalus after IVH including blockage of the arachnoid villi and arachnoid granulations due to microthrombi [61] or scarring [62]. Other theories include the CSF bulk flow hypothesis and the hydrodynamic theory. These theories are based on postulated changes in pressure gradients within the CSF or CSF and vascular systems, respectively [63, 64]. Few studies, however, have been conducted to test any of these theories.

Surprisingly, there have also been very few studies addressing the impact of IVH on the choroid plexus [65]. One recent study reports CSF hypersecretion occurs after IVH in a rodent model. This hypersecretion is caused by an upregulation in choroid plexus Na/K/Cl cotransporter activity associated with Toll-like receptor 4 activation [66].

In contrast, there is much evidence that IVH damages the ependymal cells that line the cerebral ventricles. Alterations in this CSF–brain interface may impact bulk fluid flow via ciliopathy and disrupt neurodevelopment by exposing the subventricular zone to CSF. An emerging mechanism appears to involve impaired junctional proteins, such as N-cadherin and connexin that not only provide structure for the ependymal lining of the ventricles but also influence progenitor cell proliferation, migration, and differentiation [67–70]. Recent evidence from preterm neonates with PHH also indicates that the neurogenic niche is compromised following IVH [71], and this primary effect could subsequently evolve into a type of hydrocephalus *ex vacuo,* in which the ventricles expand subsequent to impaired neurogenesis and neuronal differentiation [71].

### Current treatments for PHH of prematurity

Treatments for PHH of prematurity fall into one of three categories: temporary non-surgical, temporary surgical, and permanent surgical. Temporary non-surgical treatments, including thrombolytic agents and diuretics, have been tested [72–77]; however, none of these interventions are currently recommended for use in neonates with PHH [78]. Temporary surgical treatments include ventricular access devices (VADs), external ventricular drains (EVDs), ventriculosubgaleal (VSG) shunts, and lumbar punctures. All four temporary surgical treatments are options in the management of PHH [79]; however, lumbar puncture is only recommended for immediate short-term removal of CSF [78]. Serial lumbar punctures are not recommended [78, 80].

Emerging treatments include endoscopic lavage with early removal of IVH. In one study, Schulz et al. reported a decrease in the rates of hydrocephalus and need for permanent shunt placement [81]. However, this treatment remains under investigation and long-term outcomes remain unclear.

If hydrocephalus persists despite temporary CSF diversion, placement of a ventriculoperitoneal (VP) shunt has been the treatment of choice. VP shunting has been the mainstay of long-term surgical management of hydrocephalus for over six decades. However, VP shunts frequently fail. In children, approximately 30-50% of shunts fail within the first 2 years [82–84] and 80% of shunt fail within 4 years [83], necessitating revision, externalization, or removal, all of which have associated costs and complications. Endoscopic third ventriculostomy (ETV), with or without choroid plexus cauterization (ETV-CPC), is an alternative treatment option and can obviate the need for a shunt. However, for treating PHH, the success rate in children less than 1 year of age is approximately 50% [85–90]. Evaluations of long-term neurological outcomes of ETV-CPC or ETV versus shunt are ongoing.

## Review of clinical trials

Past clinical trials inform future clinical, translational, and basic science research. The workshop, reviewed clinical trials conducted in both premature neonates and adults with intracranial hemorrhage to identify areas of promise and obstacles that limit our ability to interpret and compare results.

### Interventions that alter risk of IVH

Premature and/or (extremely) low birth weight neonates are vulnerable populations at risk for multiple co-morbidities such as IVH and neurological deficits. To improve survival rates and decrease such co-morbidities, interventions based on a variety of biological mechanisms have been tested and adopted by the medical community. These interventions can be broadly grouped into antenatal interventions, birth-related procedural interventions, and postnatal interventions (Table 1).

**Table 1:** Summary of representative drug or physical intervention trials to reduce IVH or improve neurological outcomes in humans

Drug(s) or intervention	Dosage	Mechanism	Data class	Degree of IVH reduction	Neurological outcome improvement	References
Antenatal betamethasone	Single 2-dose (12 mg each); 24 mg	May reduce endothelial proliferation, vascular density, and increase pericyte vascular coverage [270]	Class 2, Class 2, Class 2, Class 2, Class 1, Class 2, Class 1	IVH reduction from 6 to 47 h, slowly increased 48 h ≥ 10 days); beneficial for women < 34 weeks GA; decreased IVH from 22–29 GA; decreased IVH even with incomplete course	Not significant, not examined in study; associated improved neurological outcome	[30, 91, 93, 114, 271–273]
Antenatal betamethasone/dexamethasone	Meta-study with unknown dosing	May reduce endothelial proliferation, vascular density, and increase pericyte vascular coverage	Class 2	Beneficial for women peri-viable ages 23–26 weeks GA	Not examined	[274]
Prophylactic indomethacin (oral or IV)	Oral 0.2 mg/kg daily for 3 days; 0.1 mg/kg IV for daily for 2 days	Inhibits cyclooxygenase-mediated production of prostaglandins, promotes germinal matrix vascular maturation	Class 2, Class 1, Class 1	50–80% reduction in IVH	Higher verbal scores in 3-8 yr old boys	[118, 275, 276]
Ibuprofen	Oral 5–10 mg/kg daily for 3 days	Inhibits cyclooxygenase-mediated production of prostaglandins	Class 2, Class 1	Mixed results: 0–80% reduction in IVH	Not examined	[118, 277]
Neonatal Ethamsylate	12.5 mg/kg daily for 4 days	Inhibits cyclooxygenase-mediated production of prostaglandins; possibly increases platelet aggregation	Class 1, Class 1, Class 1	Reduction in IVH	No decrease in mortality (possibly increased), no improvement in cognitive outcomes	[113, 278, 279]
Inhaled nitric oxide	Inhaled at 20 ppm	Mediates rapid vasodilation by stimulating guanylate cyclase, subsequent reduced phosphorylation of myosin, and relaxes smooth muscle cells	Class 1, Class 1	No reduction in IVH	No significant neurological improvement	[280, 281]
Phenobarbital	IV injections of 20–30 mg/kg loading dose, followed by 3–7 days of maintenance doses	Acts on GABA receptors and is thought to stabilize blood pressure and may protect against free radicals	Class 2, 3	Mixed results from 12 clinical trials; overall does not appear to reduce severe IVH or ventricular dilatation	Did not seem to attenuate neurological impairments	[282]
Prophylactic surfactant	Bolus or infusion delivery via endotracheal tube (e.g., 5 ml/kg)	Replenishes insufficient surfactant production in premature neonates in order to increase pulmonary compliance, increase alveolar gas exchange, and decrease hypoxia	Class 1, Class 1, Class 1	No reduction in IVH (though there was decrease in neonatal morbidity)	Not examined	[283–285]
Postnatal corticosteroids (typically dexamethasone)	0.12–0.5 mg/kg/day for several days	May reduce endothelial proliferation, vascular density, and increase pericyte vascular coverage	Class 1, Class 1	Possible trend towards reduction of neonatal IVH	Unclear neurological improvement	[286, 287]
Magnesium (magnesium sulfate)	4–6 g IV MgSO_4_ loading dose, 2–4 g/h for 12–24 h	Tocolytic mechanisms include competition for calcium, prevent release of acetylcholine, and activation of myosin light chain kinase, which blocks myometrial contractions; neuroprotective effects may include stabilization of rapid blood pressure fluctuations, increased cerebral blood flow, and decreased neuroinflammation	Class 1, Class 1, Class 1, Class 2	No effect on IVH reduction	Possible neurological protection	[288–291]
Cesarean delivery	22–37 weeks gestational age planned delivery	Prevention of stresses associated with vaginal delivery	Class 2, Class 2	35% reduction in IVH in < 30 week preterm neonates	Not examined	[102, 292]
Delayed cord clamping	Delayed clamping usually performed 60–75 s (compared with 30–45 s)	Proposed mechanisms include cardiovascular transition with ventilation, establishment of red blood cell volume, decreased need for blood transfusion	Class 1, Class 1, Class 3, Class 3	Up to 50% reduction in preterm IVH; benefits unclear in term neonates	May improve fine motor and social domains at 4 years in low-risk children [293]	[288, 293–295]

Antenatal interventions include administration of corticosteroids, phenobarbital, magnesium sulfate, and vitamin K. Antenatal corticosteroids administration is perhaps the most established intervention and has been shown to reduce severe grades of IVH as well as reduce mortality and respiratory distress syndrome [91, 92]. In extremely premature infants, improved outcomes in neonatal mortality and neurodevelopmental outcomes were dose dependent with an indication that these results were mediated by reduced rates of severe IVH [93]. Antenatal vitamin K administration also reduced IVH risk during the first 7 days of life in a small randomized trial [94]; however, an earlier meta-analysis concluded that antenatal vitamin K did not reduce risk of IVH or improve neurodevelopmental outcomes [95]. A recent meta-analysis indicated that, antenatal magnesium sulfate did not reduce the rates of IVH in preterm infants but may have beneficial effects on the rates of moderate to severe cerebral palsy [96]. In addition, antenatal phenobarbital had no effect on IVH risk in preterm infants (NCT00009620; [97, 98]).

Procedural interventions occurring at or close to the time of birth include mode of delivery and the timing of umbilical cord clamping. In many studies comparing vaginal and cesarean deliveries in preterm and very low birth weight infants, there did not appear to be IVH risk reduction associated with one form of delivery over the other [99–101]. By contrast, a study showed planned cesarean delivery is associated with a reduced risk of IVH in extremely preterm infants compared to vaginal delivery and emergency cesarean delivery [102]. A study comparing both antenatal corticosteroid administration and cesarean delivery reported that antenatal corticosteroid administration independently reduced IVH risk [103]. The choice of immediate/early vs. delayed cord clamping balances the desire to have neonatologists care for the preterm infant as soon as possible compared with the thought that additional blood flow from the placenta (placental transfusion) may minimize IVH and decrease the need for subsequent transfusions. An early study and a meta-review appeared to show a reduction in IVH with delayed clamping [104, 105], although subsequent primary and meta-analysis studies showed no difference in the rate of IVH [106–108]. Encouraging, there may be some neurological and motor benefits to the infants when examined after 18 months [106].

Postnatal interventions that decrease the risk of IVH, but may not improve long-term neurological outcomes, include treatment with prophylactic indomethacin, ibuprofen, inositol, or ethamsylate [31, 109–114]. Early indomethacin or ibuprofen decreased IVH rates in preterm and very low birth weight infants [115–120]. In addition, indomethacin reduced the incidence of patent ductus arteriosus and the risk of severe periventricular and intraventricular hemorrhage, although rates of shunted hydrocephalus were not changed (NCT00009646; [120]). Inositol treatment reduced the risk of severe IVH as well as neonatal deaths, infant deaths, and retinopathy of prematurity [121–125]. In a systematic review of ethamsylate treatment in preterm and very low birth rate infants, Hunt and Hey found lower rates of IVH in treated infants who were born at less than 35 weeks gestation [113]. However, there were no differences in neonatal mortality or neurodevelopmental outcomes at 2 years.

Other postnatal interventions, such as inhaled nitrous oxide, phenobarbital, prophylactic surfactant, and corticosteroids, have had no effect on IVH rates [5, 126–129] but some were associated with other positive outcomes [5, 126, 128]. In contrast, multiple interventions for premature neonates increase the risk of IVH, which include red blood cell transfusions and rapid volume expansion [130–132]. Encouragingly, vitamin E supplementation in preterm infants reduces the risk of germinal matrix/intraventricular hemorrhage in a meta-analysis of 26 studies, though there was an associated higher risk of sepsis [133].

### Neonatal interventions to stop development of progressive PHH

Pharmaceutical agents, drainage and irrigation of the ventricles, and temporizing devices have been tested to stop the development or progression of PHH after IVH. In these studies, the primary outcome is usually permanent shunt placement.

To date, pharmaceutical treatments have not been effective in stopping the development of PHH or its neurological disability [78, 134, 135]. These therapies include the use of diuretics to reduce CSF production [76, 77, 136] and fibrinolytic agents. The International Posthemorrhagic Ventricular Dilation (PHVD) Drug Trial Group tested acetazolamide plus furosemide treatment. In that trial, the combination resulted in higher rates of morbidity and did not decrease the need for a permanent shunt placement [76]. At the 1 year follow up, there was no difference in shunt placements but the group did report an increase in neurological morbidity [77]. A definitive Cochrane database review by Whitelaw et al. [43] and another study reported no difference in mortality or permanent shunt placement [78, 137]. Intraventricular thrombolytic agents, including recombinant tissue plasminogen activator (rtPA) [138], streptokinase [73, 75, 139], and urokinase [140], have been tested but are also not recommended for clinical use [78]. The two randomized studies testing streptokinase reported no difference in rates of shunt dependent hydrocephalus and also increased concerns about the development of meningitis and secondary IVH [75, 139].

Recently, a phase one clinical trial for the use of PNEUMOSTEM^®^, human umbilical cord blood derived mesenchymal stem cells, in preterm infants with grade III–IV IVH was completed. The primary outcome is unexpected death or anaphylactic shock and the secondary outcome is death or the development of shunt dependent hydrocephalus; results are pending (NCT02274428). The same group is now enrolling infants in a follow up study (NCT02673788). In related work, multiple clinical trials are underway or being analyzed to determine the effects of mesenchymal stem cells on the development of bronchopulmonary dysplasia in preterm infants (NCT01828957; NCT01897987; NCT02381366; NCT0244396).

In contrast to pharmaceutical treatments, trials that have attempted to physically wash out blood and other substances have shown more positive results [72, 141, 142]. Drainage and irrigation of the ventricles in the DRIFT (Drainage, Irrigation, and Fibrinolytic Therapy) randomized control trial did not decrease mortality or the need for permanent shunt insertion at 6 months, and the study was stopped early due to an increase the incidence of secondary intraventricular bleeding [72]. However, at 2 years, the DRIFT group had reduced rates of severe cognitive disability and death compared to the standard care group [141]. These results underscore the need for long term cognitive assessments in addition to near term outcomes such as shunt placement.

At this time, short-term temporizing procedures are often the first step in the clinical treatment of PHH, since the longevity of the condition may still be in question and, from a technical standpoint, the size of the neonate may preclude longer-term strategies such as permanent CSF shunting. Temporary surgical treatments include VADs, EVDs, VSG shunts, and lumbar punctures. Many studies have compared these treatment options but no clear results have emerged (reviewed in [78, 143, 144]). A recent prospective cohort study by the Hydrocephalus Clinical Research Network (HCRN) reported no difference in the rate of permanent shunt placement between EVDs or VSG shunts (SOPHH, NCT01480349) [145].

In future studies, morbidity, mortality, permanent shunt placement, and infection risk should all be considered as important factors. However, the timing of these interventions may have a greater impact on outcomes than the type of treatment. In one study, early treatment (based on ventricular index) with either reservoir placement or lumbar puncture was safe, effective, resulted in lower rates of permanent shunt placement, and reduced moderate-to-severe disability [146]. The results of a randomized control trial, the Early versus Late Ventricular Intervention Study (ELVIS, NCT00875758), are currently being analyzed.

### Lessons from adult intracerebral and intraventricular hemorrhage clinical trials

In adults, IVH occurs as an extension of an initial intracerebral hemorrhage (ICH) with IVH occurring in about 40% of ICH cases [147, 148]. IVH size and degree of extension through the ventricular system predict poor outcomes and mortality [11, 149, 150]. Hydrocephalus develops in two-thirds of adult IVH cases and is an independent risk factor for poor neurological outcome [11, 12]. While both neonatal and adult IVH arise from parenchymal hemorrhage, the underlying causes differ between premature neonates (rupture of immature germinal matrix blood vessels) and adults (e.g., hypertension, amyloid angiopathy, anti-coagulant use; [151]). However, information from IVH in adults may inform neonatal studies.

In adults, ICH/IVH treatment has focused on three main approaches: limiting bleeding, clot removal, and reducing secondary brain injury. Hemostatic therapy (e.g., Factor VIIa, FAST trial NCT00127283; Tranexamic acid, TRAIGE trial, NCT02625948; platelet transfusion, PATCH trial, NCT02187120) and reducing blood pressure (e.g., ATACH, NCT01176565; INTERACT, NCT00716079) have undergone clinical trials to limit continued bleeding (clot expansion) in the early phase (< 24 h) after ICH. Although some of these studies have reported statistically significant effects on limiting bleeding, there has yet to be definitive evidence of improved outcomes [152–160].

The second therapeutic approach has centered on parenchymal or intraventricular clot removal. For ICH, standard surgical evacuation has not shown statistical benefit (e.g. STICH II trial, NCT01320423) and there is interest in using minimally invasive surgery (MIS) with or without tissue rtPA to lyse the clot (MISTIE II trial, NCT01827046; ICES trial, NCT00224770; ENRICH trial, NCT02880878) [161–163]. Evidence indicates that MIS is safe but whether it improves outcomes after ICH is still uncertain [162].

As in the premature population, a number of trials for adult ICH patients with IVH have combined EVD placement with administration of thrombolytic agents. A number of systematic meta-analyses have reported decreased mortality using this combination versus external ventricular drain (EVD) alone [164, 165]. Khan et al. [165] also reported a reduction in permanent shunt placement with the combination therapy while a non-significant reduction was reported by Gaberel et al. [164]. The recent large CLEAR III trial (NCT00784134) evaluated EVD with rtPA administration vs. EVD with saline in IVH patients. There was no significant improvement in the primary outcome measure (modified Rankin Score), but there was a significant reduction in mortality compared to EVD + saline [166]. There is debate about whether the particular thrombolytic (urokinase vs. rtPA) used to remove the clot is important [164, 165]. Two subsequent meta-analyses have been performed which include the results of the CLEAR trial [167, 168]. Wang et al. [88] reported that benefit of intraventricular thrombolysis (IVT) + EVD vs. EVD alone is limited to a reduction in mortality at the expense of an increased number of survivors with moderately-severe to severe disability; subgroup analyses did not suggest an advantage of urokinase over rtPA. In a recent meta-analysis and systematic review, Baker et al. [167] reported that intraventricular thrombolytic therapy for IVH resulted in reduced mortality and potentially better functional outcomes as measured by the modified Rankin Scale or Glasgow outcome scale.

Other newer minimally invasive approaches to adult IVH studied in small clinical trials include endoscopic surgery and controlled lumbar drainage for prevention of permanent shunt dependency. A randomized trial of combined IVT with lumbar drainage vs. IVT alone for prevention of permanent shunt dependency after ICH with severe IVH was stopped following interim analysis after 30 patients due to significant efficacy of the tested intervention [169]. A meta-analysis comparing neuroendoscopic surgery combined with EVD versus combination EVD plus IVT reported significant benefits in survival, functional outcomes, hematoma evacuation rates, and need for shunt placement in favor of endoscopic techniques [170]. These promising techniques will require large-scale validation with standardized protocols.

Compared to cerebral ischemia [171], there have been relatively few clinical trials of neuroprotectants to reduce ICH/IVH-induced secondary brain injury including hydrocephalus [151, 152]. As yet, those trials have not shown any statistical benefit. Iron, from hemoglobin, is one potential clot-derived neurotoxic factor [151]. Currently, there is a phase II clinical trial for deferoxamine, an iron chelator (iDEF; NCT02175225) and minocycline (MACH; NCT01805895, [172]), an anti-inflammatory agent that also chelates iron [173]. Additional promising therapies tested in small pilot clinical trials include fingolimod, which may reduce progression of perihematomal edema [174], and transcription factor peroxisome proliferator-activated receptor gamma (pioglitazone) which augments phagocytosis and modulates oxidative stress and inflammation (SHRINC; NCT00827892) [175].

With the exception of early clot expansion, where there is little data in the neonate, there are similarities in the approaches being examined in the adult and neonate. It is hoped that information from each age group may help in devising better ways for hematoma removal and reducing the impact of clot-induced, or derived, harmful factors.

## Workshop recommendations: key areas for research and intervention

**Fig. 2:**
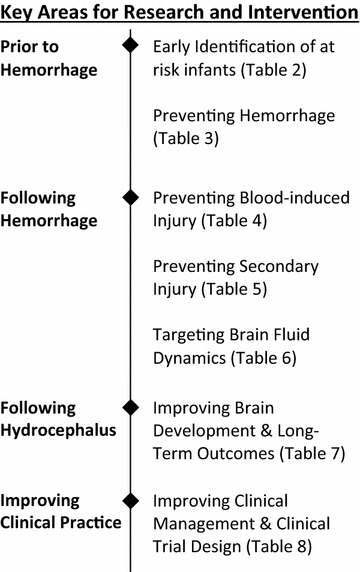
Key areas for research and intervention. A schematic of the key areas for research and intervention identified during the workshop

The development of PHH of prematurity involves a series of events resulting in multiple, simultaneous injury processes. Correctly predicting who is at risk, determining key time-points and targets for early interventions, and understanding the long-term effects of PHH are essential for preventing, minimizing, or reversing the development of hydrocephalus and improving long-term outcomes. During the workshop, key areas for research and interventions were identified and discussed (Fig. 2).

### Early identification

To optimize clinical management, it is essential to identify the premature neonates who are at highest risk of developing IVH and subsequently PHH. This is a key area for future research (Table 2). Many demographic and physiological factors are associated with IVH and PHH, but there are currently no established predictive models of PHH [176–180]. Predicting IVH and PHH with a high degree of sensitivity and specificity will improve clinical management and prognostication of high risk neonates and could be an essential component in the development and testing of new therapies. Identified biomarkers may provide insights to the pathophysiology of PHH and provide new therapeutic targets to prevent or minimize the condition. These tools could also be used as selection criteria for clinical trials testing the efficacy of new therapies.

**Table 2:** Early identification: research targets

Identifying genetic risk factors
Identifying susceptibility/risk factors of IVH and PHH
Identify diagnostic, prognostic, predictive, and monitoring biomarkers (e.g., molecular, cellular, imaging biomarkers)
Developing a prognostic scale

### Prevention of hemorrhage

Premature neonates are uniquely susceptible to germinal matrix hemorrhage (GMH) and the germinal matrix vasculature is a major target for research (Table 3). Determining the tolerance of the germinal matrix to fluctuations in cerebral blood flow and the interplay between respiratory and cardiac instability and cerebral blood flow will help guide clinical management of premature neonates to reduce the risk of GMH. In addition, a more in depth understanding of germinal matrix development and physiology will provide new targets for prophylactic therapies aimed at strengthening or otherwise modifying the germinal matrix to make it less susceptible to hemorrhage. Determining how these therapies affect corticogenesis is a critical component and potential barrier to success.

**Table 3:** Preventing hemorrhage: research targets

Determining the tolerance of the germinal matrix to respiratory and cardiac instability
Understanding the development of the germinal matrix
Understanding the physiology of the blood/ventricular barrier of the germinal matrix
Determining how to modify the germinal matrix vasculature without altering corticogenesis

### Prevention of blood-induced injury

Once IVH occurs, blood and blood-derived products cause damage to the surrounding tissue. Developing methods to reduce such injury is a major research target (Table 4). Multiple labs have already identified key blood components, such as iron and lysophosphatidic acid (LPA), that can independently cause ventriculomegaly and progressive hydrocephalus [3, 67, 181–186]. Determining the mechanism(s) of action on both the ventricular ependyma and choroid plexus epithelia may allow the development of targeted therapies, such as iron chelation, LPA receptor antagonism, or reducing LPA production. Ependymal damage after IVH may be a cause of further injury by allowing blood and blood components direct access to underlying tissue and by affecting fluid flow within the brain [71]. As stated earlier, the choroid plexus is the site of the blood–CSF barrier and the primary site of CSF secretion. Alterations in either of these functions may exacerbate brain injury and alter fluid dynamics in the brain after IVH [66].

**Table 4:** Preventing blood-induced injury: research targets

Identifying mechanisms of toxicity
Elucidating the effects of IVH on ventricular ependymal and choroid plexus epithelium
Developing targeted therapeutics
Determining how to clear the blood clot rapidly and safely

Careful time-series experimentation will be required to determine if there is a critical time window post-IVH in which these therapies are effective and if the timing can be achieved in a clinical setting. In addition, early blood evacuation with or without the use of thrombolytic agents may reduce damage and improve outcomes. Forthcoming results from the ELVIS trial should help clinical decision making. Active clearance of the blood clot remains a promising avenue for research. It may also be possible to speed up endogenous processes, such as macrophage clearance of the clot, that do not result in the release of additional blood products.

### Prevention of secondary injury

Prevention or reduction of secondary injury processes and the damage caused by hydrocephalus are active areas of research, with multiple research targets (Table 5), and may have broad implications for preventing or minimizing the effects of hydrocephalus for many etiologies. Immediately after hemorrhage, blood coagulation begins through a cascade that activates thrombin [187, 188] and results in subarachnoid fibrosis and disruption of cerebrospinal fluid dynamics [58, 189–194]. Modulation of neuro-inflammatory pathways has thus been suggested as a strategy for mitigating the effects of IVH and potentially preventing the development of PHH. CSF levels of interleukin (IL)-1β, IL-6, IL-8, IL-18, transforming growth factor (TGF)-β1 and -β2, tumor necrosis factor (TNF)-α, and other cytokines and chemokines have all been implicated in PHH [194–200]. In a recent study by Habiyaremye et al. [201], XCL-1 was decreased in the CSF of neonates with PHH. Levels of CCL-19, IL-1α, IL-1β, IL-12, and CCL-3 were elevated but only absolute CCL-19 levels correlated with levels of CSF nucleated cells, neutrophils, and lymphocytes [201]. In addition, non-protein bound iron likely remains a source of injury well after the initial bleed [3, 182, 185, 202].

**Table 5:** Preventing secondary brain injury: research targets

Therapeutic modulation of neuroinflammation
Preventing fibrosis
Preventing ependymal damage
Minimizing edema

Recent studies have focused on reducing subarachnoid fibrosis and periventricular astrogliosis by targeting inflammatory and other signaling cascades and have had positive results in animal models [194, 203–205]. Other neuroprotective attempts to modulate calcium cytopathology have shown limited improvements [134, 206, 207]. As opposed to molecular targets, the results of the DRIFT trial suggest that ventricular drainage and irrigation to remove toxic substances, such as free iron and pro-inflammatory cytokines, provides long-term benefits [141]. To date, this is the only intervention that has improved outcomes in PHH in a randomized trial.

For treatment of both blood-induced injury and secondary injuries, the specificity of the compounds, mode of administration, dosing, toxicity, and proof of target engagement will become extremely important as therapies move toward first in human trials (see section: “Moving basic science discoveries into clinical practice”). The identification of potential therapeutic agents would be accelerated if appropriate in vitro preparations were developed that could be used for high-throughput screening of new or repurposed compounds.

### Targeting brain fluid dynamics

IVH in premature neonates can result in altered brain fluid dynamics and hydrocephalus. Increased intracranial pressure (ICP) and hydrocephalus can cause additional damage through hypoxia, ischemia, and mechanical stress [58, 207–214]. This damage is especially apparent in white matter tracts [214–221]. Temporizing devices and permanent shunting can, at least partially, relieve these insults, but are only implemented after ventriculomegaly is apparent. Non-surgical methods that alter CSF dynamics could be used at earlier time points in isolation or later in conjunction with temporizing devices. Current targets for these interventions include pharmaceutical reduction of CSF production by the choroid plexus (reviewed in [134, 222]), increased CSF absorption (e.g., increase absorption through aquaporin channels [223–225], especially following IVH [226], as well as cranial nerve lymphatics [227]), and improved CSF flow (e.g., enhancement of natural processes that repair motile cilia). This list, however, also highlights the need for a better understanding of the choroid plexus response after IVH, alternative routes of CSF production and absorption, and the role of motile cilia in the development of hydrocephalus after hemorrhage. Strengthening cell–cell junctions between ependymal cells might also prevent or delay ventriculomegaly by maintaining ependymal integrity and increasing the total resistance of the ependymal layer [71, 228].

Although much research is still needed on CSF dynamics (Table 6), IVH and subsequent injury may also impact interstitial flow within the brain parenchyma. The recently proposed brain glymphatic system describes a pathway for fluid movement between CSF and brain parenchyma and within the parenchyma itself [229]. How IVH affects such movement is an important research question.

**Table 6:** Fluid dynamics: research targets

Understanding and targeting CSF fluid production
Understanding and targeting alternative routes for CSF absorption
Understanding and targeting CSF and brain interstitial fluid flow
Understanding changes in barrier function after IVH
Understanding and targeting ependymal cell–cell junctions

### Understanding brain development and long-term outcomes after IVH

Premature neonates with Grade III or IV IVH are at a higher risk of poor neurodevelopmental and cognitive outcomes than preterm neonates without IVH or who have Grade I or II IVH [7, 230]. It is not clear if the development of progressive hydrocephalus significantly adds to this burden [30], but it is likely that the injury mechanisms involved in the development of PHH overlap with those related to neurodevelopmental and cognitive outcomes.

For the PHH population, improving long-term neurodevelopmental and cognitive outcomes is very important and should be a primary focus in clinical trials [231]. Understanding how blood products, destruction of the ependymal layer, and inflammatory processes alter brain maturation will be important for the development of rehabilitation strategies. Normalized brain development and function after therapeutic interventions could serve as additional markers of efficacy beyond the primary outcome measure of permanent shunt placement. For example, recent findings of altered CSF proteins [129–131, 152] and impaired adherens junctions in disrupted ventricular and subventricular zones [71] could be targeted and have long-lasting neurodevelopmental consequences. In the near term, ventricular drainage and irrigation may minimize the secondary damage that occurs soon after and in the months following a brain bleed, minimizing the need for additional therapies [141].

Improving functional outcomes is a priority for the hydrocephalus community and must become a priority in research [231]. Longitudinal clinical studies are needed to investigate the long-term neurobehavioral outcomes of PHH, ranging from the infant/toddler stage through adulthood. For patients with shunted hydrocephalus, it is important to study the longer term, cumulative effects of altered CSF dynamics along with periodic brain compression and increased ICP due to shunt malfunction on cognition and other measures of quality of life. Other areas of particular interest include the impact of non-surgical (e.g., occupational, speech, physical therapies) and surgical (CSF shunt management) treatments. For such long-term studies, the development of optimal psychometric and imaging-based instruments is a logical priority. Research priorities in relation to brain development and long-term outcomes after IVH are given in Table 7.

**Table 7:** Brain development and long-term outcomes after IVH: research targets

Determining if PHH is associated with worse long-term neurodevelopmental, cognitive, and motor deficits compared to IVH and prematurity
Determining how damage to motile and primary cilia affects brain development
Understanding how cell proliferation from the ventricular and subventricular zones are altered after IVH
Identifying and targeting the mechanisms responsible for changes in cell proliferation
Linking neurodevelopmental and cognitive deficits to altered brain function
Developing targeted rehabilitation strategies for preterm neonates with IVH and PHH.
Determining mechanisms of tissue repair after PHH and how to enhance it
Determining the benefits of stem cell therapies on long-term outcomes
Determining impact of non-surgical interventions on long-term outcomes
Developing optimal psychometric and imaging-based instruments

### Improving clinical management and clinical trials

Focus on the clinical management of preterm neonates has intensified in recent years in parallel with advances in obstetrics and newborn medicine. Identifying and implementing best practices as well as testing the benefit, or lack thereof, of new techniques and therapies is an essential part of this process. However, in published work, substantial variability exists in the study populations as well as diagnosis and treatment thresholds, making comparisons between studies difficult.

Standardizing clinical protocols through quality improvement methodologies and/or clinical trials has become a priority for the field but more work needs to be done. An early report from the HCRN demonstrated substantial variation in the treatment of PHH, even within this coordinated network [232]. In 2014, evidence-based guidelines for the management of PHH were published and provided specific recommendations while also identifying larger areas of uncertainty [78]. Wellons et al. [145] recently published the results of the HCRN’s Shunting Outcomes in Post-Hemorrhagic Hydrocephalus (SOPHH), which compared two of the most common neurosurgical treatments for PHH, and, while no difference was found between the two treatments, SOPHH provides an excellent starting point for standardizing the diagnosis and treatment of PHH [145].

Study protocols also need to be standardized. Currently, research groups use different study populations and inclusion criteria (e.g., preterm vs. very preterm, low birth weight, very low birth weight, or IVH grade) as well as different diagnosis and treatment thresholds. As a first step, common classification data including gestational age at birth, weight at birth, and IVH grade should be collected in every study and, when possible, standard inclusion and exclusion criteria should be used.

Establishing standardized criteria for PHH diagnosis and treatment thresholds (e.g., physical criteria, ventricular size parameters, CSF and imaging biomarkers, etc.) will also help simplify the interpretation of comparative effectiveness research examining available treatments (e.g., CSF shunting versus ETV ± CPC) and studies testing emerging therapeutics. Outcome measures (e.g., ventriculomegaly, progressive hydrocephalus, symptomatic hydrocephalus, or permanent shunt placement) also need to be standardized and used consistently.

Standardizing clinical management and study protocols for preterm neonates is essential as new surgical and non-surgical strategies are developed. With powerful, multi-institutional and multi-dimensional clinical trial infrastructure, the collective efforts of committed investigators can be leveraged to address critical clinical questions relevant to the diagnosis and treatment of PHH over both the short- and long-term. Research priorities in relation to clinical management and clinical studies after IVH are given in Table 8.

**Table 8:** Clinical management and clinical trials: targets

Developing standardized management and treatment protocols
Implementing standardized management and treatment protocols
Collecting common classification data (e.g., gestational age at birth, birth weight)
Using common study inclusion/exclusion criteria when possible
Developing standardized diagnosis criteria/definitions
Developing standardized treatment criteria/definitions
Developing standardized outcome measures/definitions

## Moving basic science discoveries into clinical practice

The workshop was designed to identify areas of opportunity and spur research efforts for PHH of prematurity, but the ultimate goal is to prevent IVH and PHH of prematurity or prevent associated brain injury. The process of moving basic science discoveries into clinical trials and then standard clinical practice, however, is slow and inefficient. During the workshop, participants identified multiple areas that could be improved and opportunities to speed up the transition from basic research to clinical trials.

### Choosing the appropriate model

There is a wide range of animal models used for research in PHH of prematurity and germinal matrix hemorrhage (reviewed in [233, 234]), and no single animal model will be suitable for all biological questions. Most studies involve experimental induction of IVH in mice [67, 191, 235], rats [182, 185, 202, 236–243], rabbits [244–250], cats [251], dogs [252, 253], sheep [254], and pigs [255–258]. While the majority of these in vivo studies have employed intraventricular injection of whole blood or blood products, others have used alternative approaches [67, 247, 259–261]. In vitro models of IVH have also been used [262, 263]. Before studies begin, animal models should be reviewed to determine the model most suitable for the outcomes being measured, the clinical relevance of each model, and whether multiple animal models will be needed if the initial studies are positive [233, 234]. In addition, experimental parameters such as mode of IVH induction, intraventricular volume changes, and developmental age of animal models should be addressed when interpreting the results.

### Streamlining preclinical research

There is a paucity of studies on non-surgical therapies in PHH and one goal is to increase this number and move these therapies into clinical trial. However, the literature is littered with clinical trials that proved to be costly failures. To avoid these mistakes, it is imperative that investigators conduct preclinical research at the highest standards of scientific rigor and demonstrate robust reproducibility. It is difficult for one lab or investigator to conduct all of the experiments necessary, but it is possible to streamline these efforts through the use of standardized protocols and active collaboration. In addition, publication of negative data would benefit the entire research community.

In addition to the success or failure of an experiment or series of experiments, there are other common stumbling blocks in the development of novel treatments. Many are related to the compound itself including its safety profile, metabolism, and tissue distribution. Mode of delivery can also prevent promising therapies from moving forward. On the business side, compound manufacturing, licensing, and commercialization are issues in which many investigators are not versed but can prevent a therapy from coming to market [264, 265]. Early identification of these issues can help investigators design meaningful translational studies.

### Available resources for preclinical research

Private and public organizations provide useful resources to guide preclinical research. The Operation Brain Trauma Therapy (OBTT) consortium has published a framework for preclinical drug screening and could be a useful model for PHH research [266]. In addition, the National Institutes of Health (NIH) have developed general guidelines to improve scientific rigor and reproducibility (https://www.nih.gov/research-training/rigor-reproducibility/principles-guidelines-reporting-preclinical-research). The RIGOR guidelines and Stroke Therapy Academic Industry Roundtable (STAIR) recommendations have been developed for preclinical studies focused on stroke therapies [267–269]. These guidelines and recommendations are highly applicable to PHH research.

The National Institute of Neurological Disorders and Stroke (NINDS) has also developed programs to streamline preclinical therapeutic development (https://www.ninds.nih.gov/Current-Research/Research-Funded-NINDS/Translational-Research). For early-stage development, NINDS has created the IGNITE (Innovation Grants to Nurture Initial Translational Efforts) Program that include: (1) funding opportunities for assay development and screening potential therapeutic agents, (2) therapeutic agent characterization (pharmacokinetics, pharmacodynamics and in vivo efficacy studies for small molecules, biologics and biotechnology products and (3) the development and validation of animal models, model systems and/or pharmacodynamic markers. Additional information about this program can be found here: https://www.ninds.nih.gov/Current-Research/Research-Funded-NINDS/Translational-Research/Funding-Programs-Researchers/IGNITE.

For later-stage biologic and biotechnology products, NINDS has established the CREATE Bio (Cooperative Research to Enable and Advance Translational Enterprises for Biologics) program that includes an optimization track (agent optimizations) and a development track (IND enabling and optional small first in human Phase 1 clinical trials) funding opportunities (https://www.ninds.nih.gov/Current-Research/Research-Funded-NINDS/Translational-Research/CREATE-BIO). For later-stage drug development, NIH established the NIH Blueprint Neurotherapeutics Network (BPN) providing support for small molecule discovery and development (https://neuroscienceblueprint.nih.gov/bpdrugs/).

The Food and Drug Administration (FDA) also provides guidelines for good laboratory practices (GLP) which need to be followed for medical product development for preclinical trials (http://www.accessdata.fda.gov/scripts/cdrh/cfdocs/cfcfr/CFRSearch.cfm?CFRPart=58). Researchers should understand the guidelines and work with their institutions to establish GLP practices early in the drug development process. Resources are also available through the Alzheimer’s Drug Discovery Foundation that cover topics from central nervous system drug discovery through commercialization (https://www.scienceexchange.com/group/addf-access/resources#catList).

### Conducting clinical trials

Conducting clinical trials in PHH of prematurity present a number unique challenges. Extreme prematurity, very low birthweight, and medical comorbidities as well as often complex social issues all contribute to the vulnerability of this population. Thus, clinical studies must be held to the highest standards of good clinical practice (GCP), institutional human research protection offices, and federal agencies such as the NIH and FDA.

Despite its high prevalence within newborn medicine and hydrocephalus in general, PHH is relatively uncommon at any individual institution. Multi-institutional platforms are therefore critical to conducting meaningful human studies research in PHH. In addition to the HA-supported HCRN, the NIH has worked to develop infrastructure for clinical studies for conditions like PHH. NINDS established the Network for Excellence in Neuroscience Clinical Trials (NeuroNext) that consists of one Clinical Coordinating Center (CCC), a Data Coordinating Center (DCC), and 25 Clinical Sites located throughout the United States (https://www.neuronext.org/). The standardized and accessible infrastructure of NeuroNext was designed to efficiently and rapidly conduct exploratory phase I and II clinical trials as well as biomarker validation studies.

Through the National Center for Advancing Translational Sciences (NCATS), the NIH also has important resources for early and late phase studies designed to repurpose drugs developed for other conditions (https://ncats.nih.gov/preclinical/repurpose). For example, the Molecular Libraries Small Molecule Repository (MLSMR) has over 350,000 compounds. Taking advantage of these resources can accelerate the process of therapy development.

## Role of the Hydrocephalus Association

The HA can play a central role in accelerating hydrocephalus research by serving as a link between the two HA-supported clinical networks, the Hydrocephalus Clinical Research Network (HCRN.org) and Adult HCRN (AHCRN.org), the basic and translational researchers involved with the HA Network for Discovery Science (HANDS, HANDS.hydroassoc.org), and the patient community through the HA Patient Powered Interactive Engagement Registry (HAPPIER, hydroassoc.org/happier). HANDS is designed to support basic and translational research by directly funding research studies, building shared infrastructure, and convening workshops and conferences. HANDS is also a platform for improving communication between researchers and developing new research collaborations. HAPPIER is a patient reported database. The survey data generated through HAPPIER can be used by researchers. In addition, researchers can work with HA to send out new surveys and also to recruit patients for clinical trials. The HA will continue to develop and modify research programs to best serve the hydrocephalus research community as well as continue advocacy efforts to increase the availability of federal funding for hydrocephalus research.

## Conclusions

Intraventricular hemorrhage and posthemorrhagic hydrocephalus is a potentially devastating condition in premature neonates with no effective non-surgical therapies. The Hydrocephalus Association Posthemorrhagic Hydrocephalus Workshop identified many important areas for future research to help produce a pathway for reducing the occurrence and effects of PHH.

## Abbreviations

AHCRN: Adult Hydrocephalus Clinical Research Network
BPN: Blueprint Neurotherapeutics Network
CCC: Clinical Coordinating Center
CPC: choroid plexus cauterization
CREATE Bio: Cooperative Research to Enable and Advance Translational Enterprises for Biologics
DCC: Data Coordinating Center
DRIFT: Drainage, Irrigation, and Fibrinolytic Therapy
ELVIS: Early versus Late Ventricular Intervention Study
ETV: endoscopic third ventriculostomy
CPC: choroid plexus cauterization
EVD: external ventricular drain
FDA: Food and Drug Administration
GCP: good clinical practice
HA: Hydrocephalus Association
HANDS: Hydrocephalus Association Network for Discovery Science
HAPPIER: Hydrocephalus Association Patient Powered Interactive Engagement Registry
HCRN: Hydrocephalus Clinical Research Network
ICH: intracerebral hemorrhage
ICP: intracranial pressure
IGNITE: Innovation Grants to Nurture Initial Translational Efforts
IL: interleukin
IND: investigational new drug
IVH: intraventricular hemorrhage
IVT: intraventricular thrombolysis
LPA: lysophosphatidic acid
MIS: minimally invasive surgery
MLSMR: Molecular Libraries Small Molecule Repository
NCATS: National Center for Advancing Translational Sciences
NeuroNext: Network for Excellence in Neuroscience Clinical Trials
NICHD: National Institute of Child Health and Human Development
NIH: National Institutes of Health
NINDS: National Institute of Neurological Disorders and Stroke
PATCH: Pre-hospital Anti-fibrinolytics for Traumatic Coagulopathy and Haemorrhage
PHH: posthemorrhagic hydrocephalus
PHVD: posthemorrhagic ventricular dilation
rtPA: recombinant tissue plasminogen activator
SOPHH: Shunting Outcomes in Post-Hemorrhagic Hydrocephalus
STAIR: Stroke Therapy Academic Industry Roundtable
TGF: transforming growth factor
TNF: tumor necrosis factor
VAD: ventricular access device
VSG: ventriculosubgaleal
